# Isolation and cultivation as well as *in situ* identification of MSCs from equine dental pulp and periodontal ligament

**DOI:** 10.3389/fvets.2023.1116671

**Published:** 2023-03-10

**Authors:** Laura Beate Heilen, Jessica Roßgardt, Jutta Dern-Wieloch, Jörg Vogelsberg, Carsten Staszyk

**Affiliations:** Faculty of Veterinary Medicine, Institute of Veterinary Anatomy, Histology and Embryology, Justus-Liebig-University, Giessen, Germany

**Keywords:** hypsodont teeth, periodontium, endodontium, equine dentistry, regenerative medicine, MSCs

## Abstract

**Introduction:**

The lifelong eruption places a great demand on the dental pulp and periodontal ligament (PDL) of horse teeth. Cells within the pulp and PDL seem to play a key role during this remodeling.

**Methods:**

In this study, we isolated and cultivated MSCs (medicinal signaling cells) from dental pulp, PDL and retrobulbar fat of four horses. Subsequently, we analyzed them by flow cytometry and immunohistochemistry to determine and compare their characteristics. In addition, we localized these cells within the tissue structure *via* immunohistochemistry of histological sections. For these analyses, several surface markers were applied.

**Results:**

The described method illustrates a feasible approach to isolate and cultivate MSCs from equine dental pulp and PDL. In the flow cytometry a vast majority of cultivated cells were positive for CD90 and CD40 and negative for CD11a/18, CD45, CD105 and MHCII suggesting that these cells feature characteristics of MSCs. Immunohistochemistry of histological pulp and PDL sections showed the localization of CD90 positive cells especially in the perivascular region and the subodontoblastic layer.

**Discussion:**

Our findings indicate that the isolation and cultivation of MSCs from equine dental pulp and PDL is feasible although an elaborate and complicated harvesting protocol is required. MSCs isolated from dental pulp and PDL are regarded as candidates for new therapeutical approaches in equine dental medicine like regeneration of periodontal lesions, enhancement of periodontal re-attachment after dental replantation and stimulation of pulp-obliteration and apexification in combination with endodontic therapies.

## 1. Introduction

Dental diseases are very common in horses. Due to lifelong eruption, despite permanent mechanical load, horse teeth have to ensure the structural integrity of the periodontal ligament (PDL) and dental pulp (pulpa dentis).

The PDL belongs, together with the alveolar bone, the dental cementum and the gingiva to the periodontium, which supports the tooth (1, 2). It forms the connection between two hard substances, namely the alveolar bone and the cementum of the continuously erupting tooth. Regarding the permanent wear of the occlusal surface by a fibrous diet, this eruption of the equine hypsodont tooth is essential (3).

Corresponding to this dynamic process in the PDL, it should not be neglected that the dental pulp is also subjected to permanent remodeling. During tooth eruption, there must be a continuous production of subocclusal dentin to prevent occlusal pulp exposure. This assumes a permanent high productivity of odontoblasts. Histomorphometrical analyses, especially in the subodontoblastic layer, indicate that the equine dental pulp, unlike the brachydont dental pulp, remains lifelong in an immature, highly productive status (4). Although odontoblasts are regarded as postmitotic cells which survive lifelong, new odontoblasts, specifically after injury of dentin or odontoblasts, are regenerated from the subodontoblastic layer (5, 6).

Interestingly, cells of the dental pulp and PDL have the same genesis since both consist of mesenchymal tissue, which originates from migrating neuronal crest cells (7). Consequently, the cellular components of these tissues are derived from ectomesenchymal cells (1, 7, 8). However, blood vessels of the pulp and PDL are supposed to develop later during odontogenesis from migrating mesodermal cells (7–9).

MSCs (medicinal signaling cells) are most widely known as “mesenchymal stromal cells” or “mesenchymal stem cells”. However, we used the new term “medicinal signaling cells”, as introduced by Caplan et al. (10). Due to their supportive characteristics, which are based on immune modulation, creating trophic conditions, and regeneration we consider this term as more appropriate (11). The isolation of medicinal signaling cells (MSCs) from equine dental pulp and PDL has been described by different authors. Ishikawa et al. 2017 (12) isolated and characterized equine dental pulp stem cells from thoroughbred wolf teeth, and -Staszyk and Gasse 2007 (13) described a primary culture of fibroblasts and cementoblasts of the equine periodontium. A characterization of cells isolated from PDL was implemented for the markers CD31, pan-cytokeratin, CD90, and CD105 by Mensing et al. (14). For cells isolated from dental pulp, the expression levels of CD44, CD90, CD11a/18, CD105, MHCI, MHCII, CD34 and CD45 were evaluated (12, 15). Thereby, it must be noted that even though the ISCT (International Society for Cellular Therapy) defined some minimal criteria to be met by human MSCs (16), equine MSCs vary widely concerning their expression of surface markers. There are large differences concerning various tissues as well as individuals described (17–21).

Although the presence of MSCs has been documented in equine pulp and periodontal tissue, it remains unclear in which niche, extent, and amount the cells are present within the initial tissue. Merely Mensing et al. (14) compared MSCs isolated from gingiva, PDL and subcutaneous fat in the masseteric area, although they neither investigated the specific niche of the cells inside the original tissue nor isolated MSCs from pulp and PDL in parallel. However, a comparison between MSCs obtained from equine pulp and PDL is attractive concerning the common origin and the specific challenges MSCs face inside and adjacent to the erupting hypsodont tooth.

In this study, we aimed to develop a sustainable method to simultaneously obtain and cultivate MSCs from equine dental pulp and PDL. To this end, the cells isolated from the incisivi of four donors were cultivated and analyzed for different surface markers by flow cytometry and immunocytochemistry. These results were compared with those found for MSCs, which were isolated from the retrobulbar fat body since MSCs obtained from fat are already established as a reliable source and used for regenerative therapies. Another aim of the study was to demonstrate the specific localization of the isolated cells *in situ* by immunohistochemistry of the surface marker CD90, which was applied on histological slices of dental pulp and PDL.

## 2. Materials and methods

### 2.1. Donors

The cells were obtained from heads of four horses between the ages of 2.5–24 years (Table 1). All horses were slaughtered by a commercial butcher *via* captive bolt, followed by bleeding, due to reasons unrelated to this study. Sampling was performed within 24 h.

**Table 1:** Details of donors used for isolation.

**Donor No**.	**Age**	**Sex**	**Breed**	Time period pre-isolation^*^
**1**	2.5 y	♂	Warmblood	Fresh 1–2 h
**2**	13 y	♀	Warmblood	Cooling overnight 16 h
**3**	21 y	♀	Warmblood	Cooling overnight 24 h
**4**	24 y	♂	Haflinger	Fresh 1–2 h

* Time interval from slaughter to sampling.

### 2.2. Sampling

**Figure 1:**
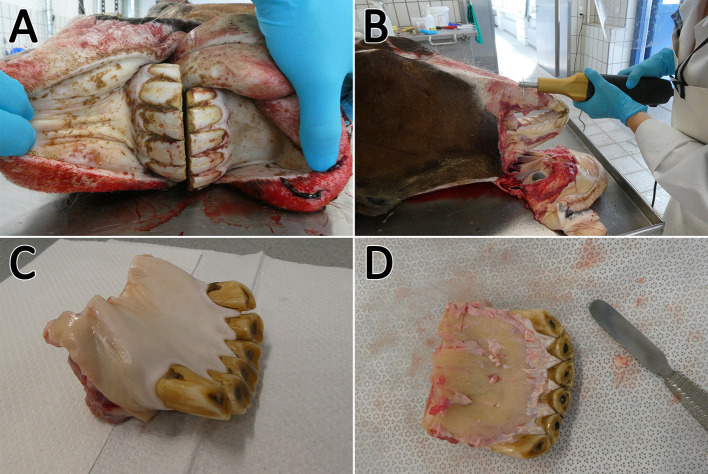
Sampling procedure. The incisors **(A)** were cleaned, the soft tissue was removed, and the arcades were dissected **(B)**. Afterwards the sample could be separated **(C)** and the remaining soft tissue was removed **(D)**. Subsequently, the sample was transferred to a microband saw.

To obtain the required tissues, the heads had to be prepared in different steps (illustrated in Figure 1). After a cleaning step with tap water, a macroscopic examination was performed to select donors with clinical healthy incisors. Due to their good accessibility, only incisors were used for cell isolation. Teeth were identified based on a tooth numbering system according to Triadan (22) and Floyd (23). Most of the soft tissue was removed from nasal up to the caudal end of the margo interalveolaris with a disinfected knife. Subsequently, the incisors were cleaned with tap water and a brush. The heads were cut with an oscillating saw through the margo interalveolaris to separate the parts of the maxilla and mandibular which include the incisors. Following a cleaning step with tap water and a short rinse with 80% ethanol, the samples were wrapped in wipes (Kimtech Wettask, Kimberly-Clark, Dallas, TX, USA) drenched with 80% ethanol and stored in the fridge at 4°C until tissue preparation for max. One hour. Before further preparation the remaining soft tissue was removed with a sterile scalpel and raspatory. For the extraction of retrobulbar fat, the skin above the retrobulbar fat body was removed with sterile forceps and scalpel. After disinfecting the forceps, scalpel, and subjacent tissue with 80% ethanol, two pieces of fat (~1 × 1 × 6 cm) were extracted. The samples were transferred to 50-ml tubes (Sarstedt AG & Co. KG, Nuembrecht, Germany) with 25 ml of transport medium B (Table 2) and stored in the fridge at 4°C for max 4 h until cell isolation.

**Table 2:** Composition of medium used for isolation and cultivation.

**Medium**	**Ingredients**	**Manufacturer**
Transport medium A	DMEM-HG^1^ 100 U/ml Penicillin 0.1 mg/ml Streptomycin 1% Amphotericin 1% Tetracycline	Life Technologies GmbH, Darmstadt, Germany Life Technologies GmbH Life Technologies GmbH Capricorn Scientific GmbH, Ebsdorfergrund, Germany Carl Roth, Karlsruhe, Germany
Transport medium B	DMEM-LG^2^ 100 U/ml Penicillin 1% Amphotericin 1% Tetracycline	Life Technologies GmbH Life Technologies GmbH Capricorn Scientific GmbH Carl Roth
Digestion medium A	Transport medium A 2 mg/ml Collagenase I 10 mg/ml BSA	Life Technologies GmbH Sigma-Aldrich Chemie GmbH, Taufkirchen, Germany Capricorn Scientific GmbH
Digestion medium B	Transport medium B 1mg/ml Collagenase I 10 mg/ml BSA	Life Technologies GmbH Sigma-Aldrich Chemie GmbH Capricorn Scientific GmbH
Cultivation medium A	Transport medium A 10% FCS 1% MEM-NEAA^3^	Life Technologies GmbH Life Technologies GmbH Life Technologies GmbH
Cultivation medium B	Transport medium B 10% FCS	Life Technologies GmbH Life Technologies GmbH

1 DMEM-High Glucose.

2 DMEM-Low Glucose.

3 MEM-Non Essential Amino Acids.

### 2.3. Tissue preparation

**Figure 2:**
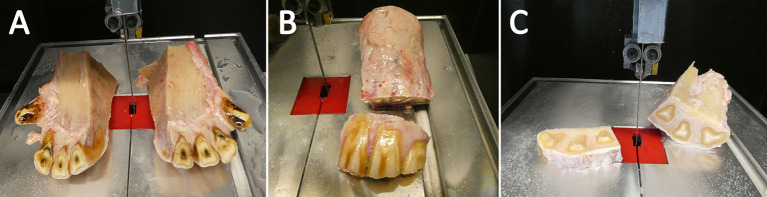
Tissue preparation. The samples were sliced through the sagittal plane **(A)**. Afterwards the clinical crone was segmented and rejected **(B)** Finally, horizontal sections were sliced **(C)**.

To isolate the dental pulp and PDL, the extracted parts of the dental arch containing the incisors had to be further dissected. After a cleaning step with tap water, a midline cut with a diamond-coated, water-cooled micro-band saw (MBS 240/E, Proxxon S.A., Wecker, Luxembourg) was performed to separate each quadrant containing teeth 01 to 03. The clinical crowns were dissected and removed before further processing. In the following, the specimens were cut into horizontal slices with a height of ~0.8 mm to isolate the dental pulp (Figure 2). For the isolation of the PDL, the sections were additionally cut through the transverse plane. After sectioning, the specimens were dipped in 70 % ethanol, washed with PBS (Life Technologies GmbH) for ~15 s, and transferred into transport medium A (Table 2). The samples were stored in the fridge at 4°C for max. Two hour until cell isolation and cultivation.

### 2.4. Cell isolation and cultivation

To avoid contamination, tissue and cell isolation occurred under sterile bench conditions. In a first step, the dental pulp and PDL had to be extracted out of the previously prepared and sectioned samples.

**Figure 3:**
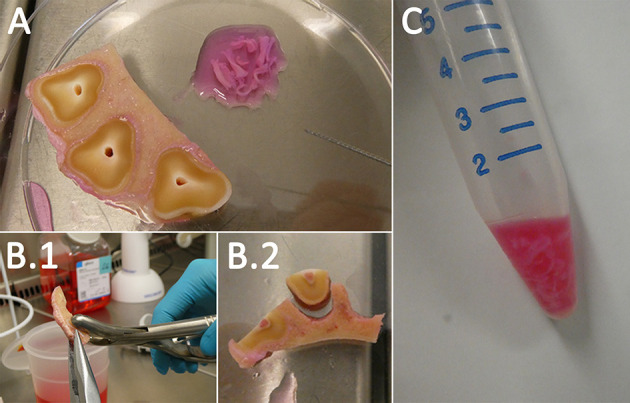
Cell isolation. The dental pulp was pulled out of its cavity *via* Hedstrom files **(A)**. To obtain the PDL the tooth and the alveolar bone were separated **(B.1, B.2)**. Afterwards, the PDL was scraped from the surfaces. Both samples were digested in 2 mg/ml Collagenase I **(C)**.

For pulp isolation, the specimen was fixed with a forceps, and the tissue was pulled out of the pulp cavity with Hedstrom files (Figure 3A). If necessary, a further dissection with a scalpel was performed. After isolation, the tissue was directly transferred in a drop of cultivation medium A (Table 2) in a petri dish.

For PDL isolation, the smaller additional transversal sectioned samples were used. The specimens were fixed with a pincer on the alveolar bone, and a rongeur forceps was used to rupture the tooth (Figure 3B). Subsequently, the exposed PDL could be scraped from the alveolar bone and cementum of the tooth with a rongeur forceps. If necessary, further dissection with a scalpel was performed. After isolation, the tissue was directly transferred in a drop of cultivation medium A (Table 2) in a petri dish. The extracted dental pulp and PDL were collected in 15-ml tubes (Sarstedt AG & Co. KG), and the same volume of digestion medium A (Table 2) was added. To digest the tissue, the tubes were transferred in a water bath at 37°C for ~60 min while agitating the suspension occasionally. Following digestion, the suspension was centrifuged at 300 *g* for 5 min, and the detached pellet with some of the supernatant above (~2 ml) was filtered through a 70-μm cell strainer (Sarstedt AG & Co. KG). The tissue remaining in the filter was directly seeded in 25-cm^2^ (Sarstedt AG & Co. KG) cell culture flasks with cultivation medium A (Table 2). Additionally, the flow-through was centrifuged at 300 *g* for 5 min and, after discarding the supernatant, resuspended in 1 ml of cultivation medium A (Table 2) and seeded in 25-cm^2^ cell culture flasks.

Afterwards, the MSCs from retrobulbar fat were isolated as previously described by Pascucci et al. (24) for MSCs isolated subcutaneous of the region above the dorsal gluteal muscles of horses. Briefly, the collected fat was cut into pea-sized pieces and the same volume of digestion medium 2 (Table 2) was added. Digestion was implemented for 40–50 min at 37°C while occasionally agitating the suspension. Following digestion, the suspension was centrifuged at 300 *g* for 5 min, and the detached cell pellet was filtered through a 70-μm cell strainer. The flow-through was again centrifuged at 300 *g* for 5 min and, after discarding the supernatant, resuspended in cultivation medium B (Table 2), and seeded in 75 cm^2^ cell culture flasks (Sarstedt AG & Co. KG).

Cells from all sources were cultivated at 37°C in a humidified atmosphere of 5% CO_2_ and 95% air. When they reached 80% confluence, they were passaged up to passage 3, which was used for further investigations. Only for immunohistochemistry, the cells were cultured up to passage 4. After one week of cultivation, supplementation of amphotericin and tetracylcine was stopped. The cell morphology of each passage was determined under phase contrast light microscope (Leica DM IL, Leica Microsystems GmbH, Wetzlar, Germany).

### 2.5. FACS-analysis

Fluorescence-activated cell sorting (FACS) analysis was performed to characterize the MSCs and compare MSCs isolated from different extraction points. Since there are no standardized criteria for equine MSCs, we decided to apply common markers as previously described by different authors for the horse [e.g., (25, 34, 39)]. Hence, CD11a/18, CD45, CD44, CD90, CD105, and MHCII were applied as markers. The FACS analysis was performed as described before by Leisengang et al. (26). We also tested the proportion of live cells by, staining with 7-amino-actinomycin D (7-AAD, Becton Dickinson, Heidelberg, Germany). For this, 7-AAD was added in a concentration of 1:50 to the cell suspension and incubated for 10 min in the dark. A list of applied antibodies can be found in Table 3. Finally, the measurement of the resuspended pellets was implemented with the FACS BD Accuri™ C6 (Becton Dickinson), and the evaluation was conducted using the BD Accuri™ C6 Software version 1.0.264.21.

**Table 3:** Antibodies for FACS-anaysis.

**Name**	**Manufacturer**	**Dilution**
**Primary antibodies**		
Rat anti-mouse CD 44 Clone: IM7, Cat No.: 553131	BD Bioscience	1:400
Mouse anti-human CD 45 Clone: UCHL1, Cat No.: 304202	BioLegend GmbH, Koblenz, Germany	1:100
Mouse anti-human CD 90 Clone: 5E10, Cat No.: 5555593	BD Bioscience	1:400
Mouse anti-human CD 105 Clone: SN6, Cat No.: 14-1057-82	eBioscience™, Thermo Fisher Scientific	1:500
Mouse anti-horse MHC Class II Clone: CVS20, Cat No.: 1085ga	BioRad, Muenchen, Germany	1:200
**Secondary polyclonal antibodies**		
PE goat anti-mouse Ig Cat No.: 550589	BD Bioscience	1:800
APC goat anti-rat Ig Cat No.: 551019	BD Bioscience	1:600
**Conjugated primary antibody**		
FITC mouse anti-horse CD 11a/18 Clone: CVS9, Cat No.: MCA1081	BioRad	1:200
**Isotype control**		
normal mouse IgG-FITC Cat No.: sc-2339	Santa Cruz Biotechnology, Inc., Heidelberg, Germany	1:100

### 2.6. Immunocytochemistry of isolated cells

Immunocytochemical analysis of the isolated and cultivated cells should show the expression of the markers CD90 and CD44, in addition to the histological sections, which should illustrate the localization of the cells inside the tissue structure. The MSCs in passage 4 were cultured on chamber slides (8-well, Sarstedt AG & Co. KG) until they reached 80% confluence. Subsequently, they were fixed with 1:1 methanol/acetone (1:1, Carl Roth), cooled to −20°C for 1 min and dried with a dryer. Afterwards, the samples were rehydrated three times for 5 min with PBS. This step was followed by blocking with a blocking solution (5% goat serum in PBS with 0.1% Tween) for 30 min and a washing step with PBS-Tween (0.1% Tween) for 3 min. The samples were incubated with the primary antibody (Table 4) overnight at 4°C in a humidity chamber, followed by three times washing for 5 min and incubation with the secondary antibody (Table 4) in the dark for 1 h. Subsequently, the antibody was washed out twice for 5 min, and the samples were incubated with 2-(4-amidinophenyl)-1H-indole-6-carboxyamidine (DAPI; Life Technologies GmbH; 1:20,000) for 2 min in the dark to stain the nuclei. After another washing step twice for 2 min, the slides were mounted with ibidi mounting medium (ibidi GmbH, Martinsried, Germany) and finally examined with the Zeiss Axio Observer Z.1 (Carl Zeiss, Göttingen, Germany). For negative controls, the samples were incubated with secondary antibody to exclude non-specific binding.

**Table 4:** Antibodies for immunohistochemistry.

**Name**	**Manufacturer**	**Dilution**	**Application**
**Primary antibodies**			
Rat anti-mouse CD 44 Clone: IM7, Cat No.: 553131	BD Bioscience	1:100	On cells
Rabbit anti-mouse CD 90 Clone: D3V8A, Cat No.: 13801	Cell signaling, Danvers, MA, USA	1:700	On cells and on tissue slices
**Secondary polyclonal antisera**			
Rabbit F(ab')2 anti-rat IgG:FITC (Code: STAR17B)	BioRad	1:200	On cells
Cy3-conjugated Affinipure donkey anti-rabbit, polyclonal IgG (H+L) (Code: 711-165-152)	Jackson ImmunoResearch, West Grove, PA, USA	1:400	On cells and on tissue slices

### 2.7. Immunohistochemistry of histological sections from dental pulp and PDL

To find the specific niche of the isolated MSCs inside the tissues, immunohistochemistry of CD90 was implemented on histological sections of dental pulp and PDL.

**Figure 4:**
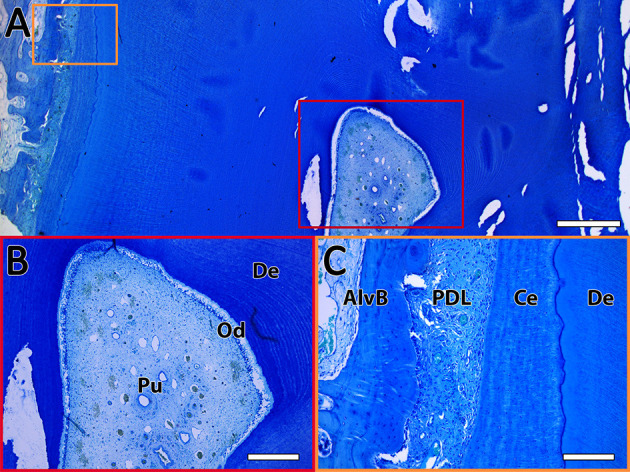
Exemplary toluidine blue staining of a horizontal sectioned incisor. **(A)** Overview of the dental pulp (red rectangle) and the PDL (orange rectangle). The scale bar represents 1,000 μm. **(B)** Enlarged view of the dental pulp (Pu) with the odontoblastic layer (Od) surrounded by dentin (De). The scale bar represents 400 μm. **(C)** Enlarged view of the PDL between the alveolar bone (AlvB) and the cementum (Ce) followed by the dentin (De). The scale bar represents 200 μm.

During tissue preparation for cell-isolation, additional section planes of the incisors were cut and fixed in 10% buffered formalin (pH 7). After fixation, the sections were watered, further trimmed with a diamond-coated, water-cooled micro-band saw, and decalcified on a platform shaker (Polymax 1040, Heidolph Instruments, Schwabach, Germany) in buffered EDTA (ethylenediaminetetraacetate) for 6 weeks as previously described by Roßgardt et al. (27). Following this decalcification process, the samples were trimmed using a scalpel to minimize section size but receive both PDL and pulp within one section. Afterwards, they were placed in embedding cassettes (Simport™ Acetal Macrosette, Fischer Scientific GmbH, Schwerte, Germany) and decalcified in EDTA for another 2 weeks. Subsequently, the samples were rinsed in tap water and stored in PBS overnight. Paraffin embedding, sectioning, and staining were implemented according to Roßgardt et al. (27). Toluidine blue staining was performed according to a standard protocol to evaluate the sections under a light microscope (Leica DM2500, Leica Microsystems GmbH). After the evaluation (example see Figure 4), paraffin sections which included both PDL and pulp were selected and incubated for 30 min at 60°C on a heating plate (MSH-20D, Witeg, Wertheim, Germany) to promote adhesion on the slides (SuperFrost Plus™, Fischer Scientific GmbH). This step was followed by de-waxing the samples in a descending alcohol series and a pre-treatment in heated citrate buffer (pH 6) at 70°C for 2 h. Afterwards, the slides were washed three times for 2 min in PBS-Tween and blocked for 30 min with a blocking solution (5% goat serum in PBS with 0.1% Tween). Incubation with the primary antibody (Table 4) was implemented overnight at 4°C in a humidity chamber, followed by washing three times for 2 min in PBS-Tween. Subsequently, the samples were incubated with the secondary antibody (Table 4) in the dark for 1 h and washed three times for 2 min with PBS-Tween. To stain the nuclei, the specimens were incubated with DAPI for 2 min in the dark and afterwards washed twice for 2 min. Finally, the slices were mounted with ibidi mounting medium and examined with the Zeiss Axio Observer Z.1 (Carl Zeiss, Göttingen, Germany). For negative controls, the samples were only incubated with secondary antibody to exclude non-specific binding.

### 2.8. Statistical analysis

The results of the FACS analysis were plotted using GraphPad Prism 6 (GraphPad Software, Inc., La Jolla, CA, USA) and evaluated using SigmaStat 4.0 (Systat Softwares Inc., San José, CA, USA). A one-way analysis of variance with repeated measurements was applied to look for similarities in marker expression. The repetition of measurements was given by the different tissues of each donor. However, it must be considered that the number of donors was low, and thus, the power of the performed test was, in most instances, below the desired power. All data are expressed as mean ± SD.

## 3. Results

### 3.1. Cell isolation and cultivation

**Figure 5:**
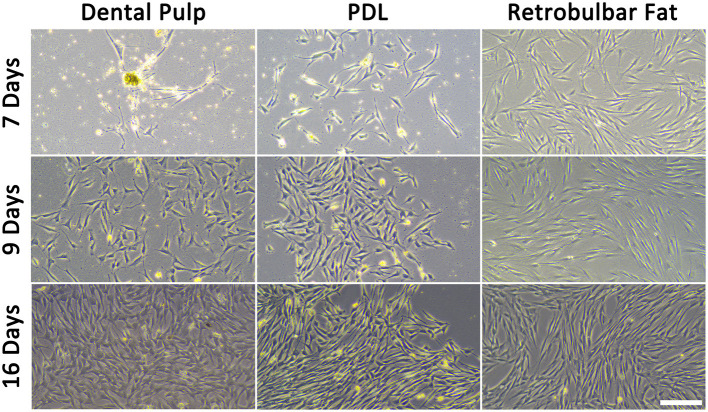
Isolated MSCs after different time points. The scale bar represents 200 μm.

The applied method was suitable to isolate viable and proliferating MSCs simultaneously from the equine dental pulp and PDL of the same tooth. Vital cells from all different tissues of the four donors were successfully isolated and cultivated (Figure 5). Donors and tissues varied in the time the cells needed to attach and become confluent. If the supernatant which remained in the cell strainer was directly seeded, cells attached (~2 d) and became confluent (~5 d) earlier, as if the flow-through was seeded. Due to their low quantity, it was not possible to obtain cells from the flow-through of donors 2 and 4. In Table 5, the different time periods of attachment and first passaging are listed for the different donors and tissues. Only the earliest time points are presented, which was constantly the case when seeding the remaining tissue inside the cell strainer. In general, most cells were gained from the retrobulbar fat and the fewest from the dental pulp. The youngest donor was attaching and proliferating much faster as a considerably higher amount of material was obtained. The MSCs needed 1–6 days to attach, and the first passaging was possible after 1–16 days. Thereby, cells gained from retrobulbar fat mostly attached early and therefore were passaged first, in contrast to cells isolated from the dental pulp, which generally needed more time. However, no difference in cell growth was detected when the time from slaughter to sampling was expanded up to 24 h. Furthermore, the donors aged between 13 and 24 years showed no obvious increased proliferation or decreased cell numbers.

**Table 5:** Time periods MSCs needed to attach and get confluent, when seeding the remaining tissue inside the cell strainer.

**Donor No**.	**Age**	**tissue**	**attaching**	**First passaging (cells obtained)**	Time period pre-isolation^*^
1	2.5	PU PDL Fat	1 d 1 d 2 d	1 d (sep.)^1^ - 10 d P1^2^ (3.15 Mio) 1 d (sep.) - 10 d P1 (9.4 Mio) 10 d (8.2 Mio)	Fresh 1–2 h
2	13	PU PDL Fat	6 d 6 d 6 d	16 d (1.6 Mio) 16 d (0.3 Mio) 13 d (5 Mio)	Cooling overnight 16 h
3	21	PU PDL Fat	3 d 3 d 2 d	15 d (0.7 Mio) 10 d (1 Mio) 8 d (4 Mio)	Cooling overnight 24 h
4	24	PU PDL Fat	4 d 4 d 3 d	16 d (0.9 Mio) 12 d (3.3 Mio) 12 d (3.8Mio)	Fresh 1–2 h

* Time interval from slaughter to sampling.

1 Separation of cells since they were growing too dense.

2 Passage 1 after 10 days.

### 3.2. Morphology of MSCs

**Figure 6:**
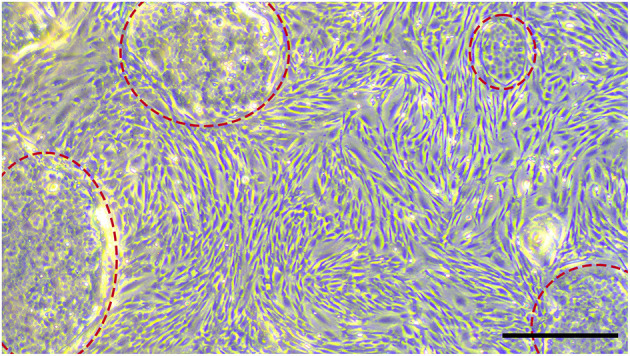
MSCs isolated from the youngest donor (2.5 years) mixed with a subpopulation of cells, which formed cobblestone-like clusters (highlighted with red circles). Scale bar represents 500 μm.

To prove if the cells had features of MSCs, initially, the morphology was evaluated. The cells grew plastic-adherend and showed an MSC-like spindle-shaped morphology, except for some isolates from the PDL of the youngest donor (donor 1), which had polygonal, large, flattened cell bodies. When growing denser, these cells formed some cobblestone-like clusters (Figure 6).

### 3.3. FACS-analysis

**Figure 7:**
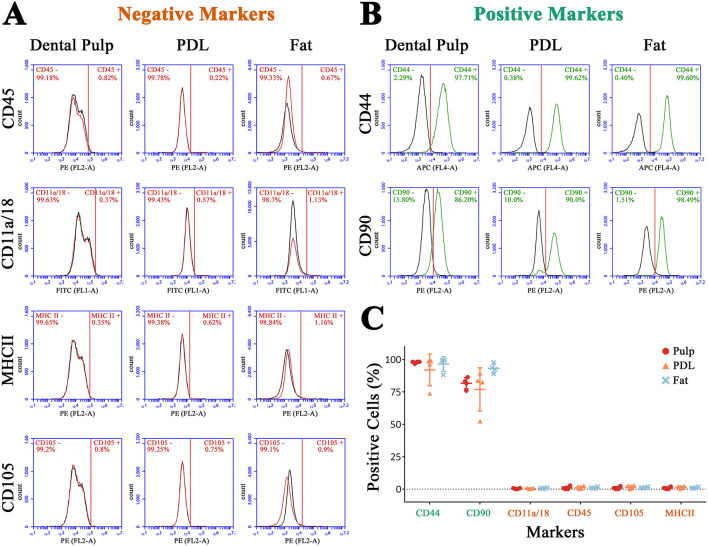
Flow cytometry for negative markers **(A)** and positive markers **(B)** of one donor exemplary. In **(C)** the ratio of positive cells for all donors is shown.

In addition to the evaluation of cell morphology, the surface marker content was analyzed by flow cytometry. Most cells were positive for CD44 (95.53% ± 7.17) and CD90 (83.96% ± 11.11) and negative for CD11a/18, CD45, CD105, and MHCII (Figure 7C). Referring to the mainly negative surface marker, no marker was expressed by more than 2.8% (CD105, donor 2, PDL) of cells. In Figures 7A, B, the marker expression of one donor is shown exemplary. Between the extraction points, no statistical differences were detected. Almost all cells isolated from pulp tissue (97.8% ± 0.76), PDL (92% ± 12.21), and fat (96.55% ± 5.55) were positive for CD44. However, for CD90, less cells showed a positive expression. For cells extracted from pulp, on average, 81.8% ± 4.32, from PDL, 77% ± 16.67, and from fat tissue, 93.15% ± 3.61 were positive for CD90. The MSCS isolated from the PDL of donor 2 showed a lower CD44 and CD90 expression than others, an early detachment from the plastic surface, and high rates of cell death after passage 3. However, it was conspicuous that MSCs extracted from dental pulp and retrobulbar fat showed a tendency to be more homogenous than those isolated from the PDL (Figure 7C).

The ratio of living cells decreased from retrobulbar fat (98.23% ± 1.19) to PDL (95.78 ± 4.11) to pulp (85% ± 8.07), without a statistical significance (for detailed data, see Supplementary Figure 1).

### 3.4. Immunocytochemistry of isolated cells

**Figure 8:**
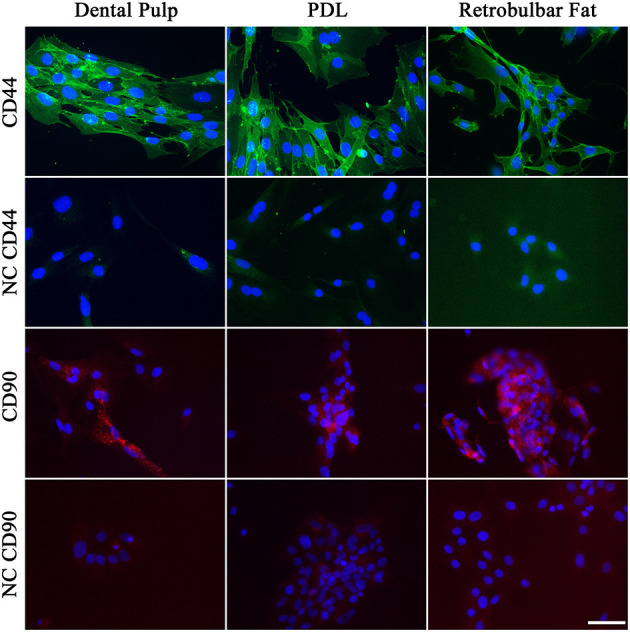
Immunohistochemistry of MSCs for CD44 (green) and CD90 (red). The nuclei were stained with DAPI (blue). Negative controls (NC) are shown beneath the sample. Scale bar represents 50 μm.

Immunocytochemistry of the MSCs was performed to evaluate the morphology of cells positive for CD44 or CD90 (Figure 8). As shown in Figure 8, the MSCs of all three tissues showed a strong positive signal for CD44, which was evenly distributed throughout the cell. The cells showed a more flattened polygonal morphology compared to routine cell cultures, due to their low density in the chamber slides.

The signal for CD90 was missing in some cells, which matches the results of the flow cytometry, where a subpopulation of cells was negative for CD90.

### 3.5. Immunohistochemistry

**Figure 9:**
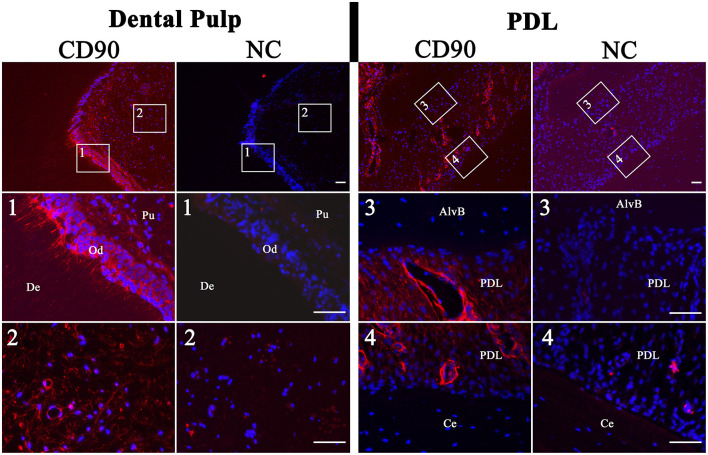
Immunohistochemistry of dental pulp and PDL for CD90 (red). The nuclei were stained with DAPI (blue). Associated negative controls (NC) are pictured besides the samples. The dental pulp showed a high-intensity signal of the odontoblastic layer (Od). Even inside the longitudinally broached dentine tubuli (De) and within the dental pulp (Pu) a signal was detected. The PDL showed a diffuse signal and a more intense perivascular signal. Cells inside the alveolar bone (AlvB) and cementum (Ce) were negative. Scale bar represents 50 μm.

The immunohistochemistry of tissue slices demonstrated the location of CD90-positive cells inside the tissue structure (Figure 9). In the dental pulp positive cells which showed a weak signal could be found diffuse inside the tissue. Cells with a high-intensity signal were localized peripheral in the area of the subodontoblastic layer. Also, the odontoblasts inside this area showed a strong signal for CD90. This was especially seen inside longitudinally broached dentine tubuli, where the odontoblastic extensions are located. Inside the PDL, perivascular localized cells with an intense signal were found. However, cells inside the PDL tissue showed a weaker diffuse signal, which was similar to those inside the dental pulp. No signal was detected in cells inside the alveolar bone and cementum.

## 4. Discussion

We established a potential method to isolate MSCs from equine dental pulp and PDL simultaneously out of the same tooth. Furthermore, we demonstrate the localization of these cells *in situ*. The obtained cells of all donors were viable and showed important characteristics of MSCs, such as a spindle-shaped morphology and a characteristic content of surface markers, e.g., CD90 and CD44. In addition, we demonstrate the diffuse particular perivascular location of CD90-positive cells inside the PDL and their diffuse, more specifically subodontoblastic location, inside the dental pulp.

### 4.1. Cell isolation and cultivation

During cell cultivation, the variations in time the cells needed to attach and become confluent were conspicuous. Above all, cells attached and proliferated faster when the supernatant in the cell strainer was seeded instead of the flow-through. It can be assumed that the cells in the supernatant are partially still integrated in the tissue structure, and hence, attachment and proliferation might be easier, compared to when the cells are separated in the flow-through. Furthermore, it is possible that more cells remain in the cell strainer than are filtered. Most authors describe the isolation of dental MSCs for other species after filtration through a 70-μm^2^ cell strainer to reduce extracellular components [e.g., (28, 29)]. Possibly, after filtration, the purity of the cell population is higher. However, during the isolation of equine dental MSCs, commonly, no filtering step is included (12, 14, 15), and our results indicate that a suitable cell population can be easily gained without filtering.

Most cells were gained from the retrobulbar fat and fewest from the dental pulp. This might be due to the high amount of fat tissue, which was easily obtained, instead of the small portion of PDL and dental pulp (Figure 4). From the youngest donor (2.5 years), more PDL and pulp tissue could be gained than from the donors aged 13–24 years. Subsequently, less cells were isolated from aged donors, and the cells needed more time to attach to the surface and grow confluent. Because of the eruption of the tooth and therewith reduction of especially pulpal tissue, fewer material might be obtained from older donors (30, 31). In addition, the pulpal cavity is steadily filled up with new layers of dentine and is therefore constantly becoming smaller (32). Schrock et al. (31) describe a starting reduction of incisor length at an age of 13–15 years. Since we found no obvious difference in cell growth in horses aged 13–24 years, a key factor for the reduction of obtained pulpal tissue in aged horses might be their smaller pulp cavity (27, 32). Even the conditions of the teeth and donor seemed to play a role since an infected tooth or a sepsis due to colic caused bacterial contamination during cultivation. Due to their resistance, it was important to avoid any contamination with yeasts, which are sometimes located in the oral cavity (33). Furthermore, the surrounding hard substances hamper the isolation of the soft tissue of teeth. Nevertheless, even using donors older than 20 years, it was possible to isolate viable cells, despite the reduction in pulpal tissue with increasing age (32).

### 4.2. Morphology of MSCs

Some cells isolated from the PDL of the youngest donor formed cobblestone-like clusters. This effect might be due to the impurity of cementoblasts, which are adjacent to the PDL and might easily get access to the culture during cell isolation. Because of the different condition in younger teeth, were the PDL detaches very easily together with cementoblasts from the teeth it is quite possible that even in younger donors cementoblasts are transferred into the culture. Staszyk and Gasse (13) describe equine cementoblasts as solitary flattened wide cells which build cobblestone-like clusters with increasing density. The authors identified cementoblasts by a missing expression of alkaline phosphatase, smooth muscle actin, and pro-collagen. As we obtained high amounts of similar cells in cultures of the youngest donor, we also cannot exclude the presence of epithelial cells derived from the enamel organ. Further investigations should, for example, include the detection of pan-cytokeratin to identify epithelial cells. Nevertheless, it is more likely that during the extraction of PDL, parts of the cementum remained adherend to the PDL and afterwards were displaced to the culture. Thus, for younger donors an adjustment of the isolation method is required. One ought to make sure that the scraping with the bone rongeur forceps is only implemented on the side of the PDL which is adjacent to the alveolar bone.

### 4.3. Cell surface marker

CD44 is largely applied and described as a positive marker for MSCs (12, 14, 15, 34), whereas CD11a/18 and CD45 are classified as negative markers (14, 15). This fits our finding as an average of 97.79% ± 0.59 of MSCs isolated from dental pulp and 92% ± 9.46 isolated from PDL expressed CD44. CD11a/18 was expressed by < 1% of cells from dental pulp and PDL and CD45 by <2.6%. For CD90, a subpopulation of cells was negative, which supports the heterogeneity of this marker. Barberini et al. (19) reported that 67.7% of MSCs isolated from the umbilical cord of horses expressed CD90, in contrast to other studies where the level was higher. According to Paebst et al. (21), MSCs isolated from adipose tissue showed the highest expression of CD90. This is consistent with our finding that MSCs from the retrobulbar fat body showed the highest expression of CD90. Nevertheless, in their study, only 24.4 ± 14.43% of cells isolated from fat tissue were positive for CD90. Our findings showed a substantially higher expression of 93.15 ± 2.8% of cells from the retrobulbar fat body. Unfortunately, in the literature, there are no quantities given for the expression of CD90 by MSCs isolated from equine dental pulp and periodontium; it is only reported that the expression is high (12, 14). Some authors describe CD105 as stemness marker (12) and some do not (20). In general, the expression of surface-markers is varies depending on the source (21) and even the individuum. In particular, MHCII expression is described as being inhomogeneous among different breeds and individuals (35, 36). In this study, the expression of all donors was <2.5%, which might later promote an allogenic application (36). The MSCs isolated from PDL appeared to be most inhomogeneous, which might be due to the proximity to the cementum, which promotes the impurity with cementoblasts. Nevertheless, only one donor showed a heterogeneous cell morphology and it should be taken into account that the surface marker expression of MSCs isolated from different equine tissues is quite various (21).

The descending rate of living cells from retrobulbar fat to PDL to pulp might be due to the greater senescence of MSCs isolated from PDL and dental pulp. The smaller number of cells obtained particularly from the dental pulp might lead to a faster senescence, on account of the required higher proliferation to become confluent. However, this tendency was not significant, and more donors ought to be analyzed.

### 4.4. Immunohistochemistry

The immunocytochemistry of the cells largely fits the flow cytometry. Most of the cells were positive for CD44. Although the signal for CD90 was missing in some cells, most cells were positive. This matches the results of the flow cytometry, where a subpopulation of cells was negative for CD90.

In addition, the immunohistochemistry of dental pulp and PDL clearly showed CD90-positive cells.

Inside the dental pulp, these cells were mainly found in the subodontoblastic layer, which is also described for rat by Hosoya et al. (6). This might support the theory that undifferentiated cells, which can differentiate toward odontoblasts, are provided by the subodontoblastic layer (37, 38). There was a remarkably strong signal of odontoblasts, even inside their processes, which has not been reported yet. Sano et al. (38) describe no immunoreactivity for CD90 in odontoblasts adjacent to the predentin in brachydont teeth of rats. However, after the authors had performed a cavity preparation, odontoblasts and subodontoblastic cells were disarranged and vanished, and the CD90 expression decreased after 1 day of preparation and increased again after 5 days. This result indicates that CD90-expressing cells play a role in the regeneration of subodontoblastic cells and odontoblasts (38). In addition, Hosoya et al. (6) describe an absent immunoreactivity for CD90 in odontoblasts of rat molars and incisors. Furthermore, it seems that CD90 is not expressed in the early phase of odontoblast development and thus appears later, when differentiation is proceeding. Until now, there is no description of CD90 expression inside the equine dental pulp. However, in the odontoblasts of rat brachydont molar and hypselodont incisors, CD90 expression seems to be missing (6). This is in contrast to our findings for the equine hypsodont tooth. In the study of Sano et al. (38), some CD90-positive cells in the superficial odontoblastic layer were found 3 days after cavity preparation. This might lead to the assumption of a steady process of lifelong odontoblast regeneration in unaffected equine teeth, which resembles the process in brachydont teeth after cavity preparation. Furthermore, the results support the findings of Roßgardt et al. (27) that the equine dental pulp remains lifelong in an immature highly productive status and contains a subodontoblastic supportive zone to ensure the continuous production of dentin.

In the PDL CD90-positive cells were localized perivascular, showing a strong signal and diffuse inside the tissue with a weaker signal. Esteves et al. (39) reported that MSCs might originate from pericytes and retain some of their features in culture. This promotes our finding of perivascular cells with a strong positive CD90 expression, which might reach the cell culture. Zhao et al. (40) describe perivascular-associated CD90-positive cells inside the PDL of mice. They experimentally induced periodontitis of the upper second molars and found out that CD90-positive cells recover their ability to form cementoblasts under these conditions. Additionally, an increase in mechanical force seems to reactivate the C90-positive cell population to differentiate to cementoblasts. This process might also be found in the PDL of unaffected equine teeth permanently exposed to mechanical force by eruption and dental wear. Thus, we detected large amounts of perivascular CD90-positive cells inside the PDL of the hypsodont equine tooth in contrast to the condition in brachydont molars of adult mice (40).

As expected, the cells inside the cementum and alveolar bone showed no immunoreactivity for CD90 since they were differentiated cementoblasts and osteoblasts.

### 4.5. Conclusion

We developed a feasible method to isolate and cultivate cells from equine dental pulp and PDL. Our evaluation of morphology and surface marker content indicated that the obtained cells possessed features of MSCs. Although standardized criteria for equine MSCs are missing, not least because of their inhomogeneity, the odontoblastic and subodontoblastic localization of CD90-positive cells inside the dental pulp is an indicator for the lifelong remodeling since in brachydont teeth, CD90 is missing in the odontoblastic layer (6). The perivascular localization of CD90-positive cells inside the PDL is a hint that the MSCs originated from pericytes (11). Furthermore, there are larger amounts of these perivascular cells than described for molars of adult mice (40). Both the equine dental pulp and PDL show adjustments to the permanent dental wear and eruption by the alteration of CD90-positive cells, which play an important role during cell differentiation. These findings suggest that equine MSCs inside dental pulp and PDL are promising for further approaches to understand the processes in horse teeth during eruption, providing an opportunity for future starting points in equine dentistry.

### 4.6. Limitations of the study

One of the most important limitations of this study is the small sample size. To make clearer statements regarding the impacts of donor age and tissue source on the MSCs, a larger sample size is required. Nevertheless, the results show that the described method can be used to isolate viable MSCs from equine dental pulp and PDL of the same teeth, even if the donor is aged.

Especially when regarding its high expression rate, CD44 seems to be a better immunohistochemical marker for the localization of MSCs inside the tissues. Unfortunately, we found no well-performing CD44 antibody for immunohistochemistry. However, many authors describe parallels of pericytes *in vivo* with MSCs *in situ* [e.g., (11, 41–43)]. This fits our findings of strong CD90-positive cells inside the perivascular region of the PDL. In dental pulp, it seems that the odontoblastic and subodontoblastic layers are a source for MSCs. This finding is in line with the high remodeling rate inside this area (44).

## Data availability statement

The original contributions presented in the study are included in the article/Supplementary material, further inquiries can be directed to the corresponding author.

## Ethics statement

Ethical review and approval was not required for the animal study because all horses included in the study were slaughtered on ground unrelated to the study. Due to this no ethical approval was required. The associated kTV number given by the regional council is 19 c 20 15 h 02 Gi 18/17 kTV 5/2021.

## Author contributions

LH, JR, and CS were responsible for conceptualization and study design. LH, JR, JD-W, JV, and CS performed the experiments. Original draft preparation and revision was conducted by LH and CS for visualization LH and for supervision CS was responsible. All authors have read and agreed to the published version of the manuscript.
