# Supplementary figures and images for: Isolation and cultivation as well as in situ identification of MSCs from equine dental pulp and periodontal ligament
Source: Front Vet Sci. 2023 Mar 10;10:1116671. doi: 10.3389/fvets.2023.1116671 (PMC10036573; doi:10.3389/fvets.2023.1116671)

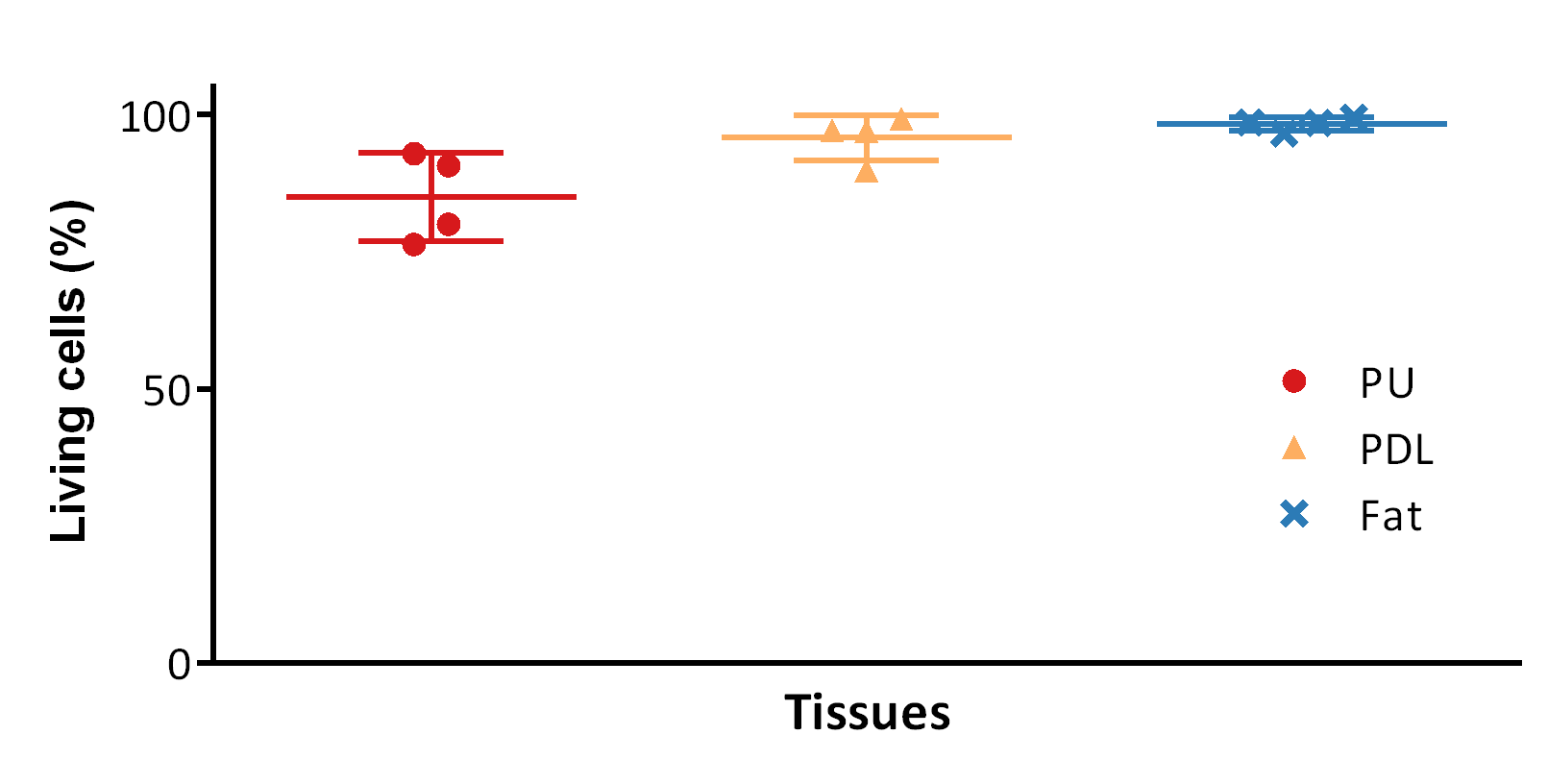

Supplement: Supplementary file 1 [file Image_1.tif]
